# Prediction of Amyloid Status using EEG‐Based Brain Effective Connectivity

**DOI:** 10.1002/alz70856_104038

**Published:** 2025-12-24

**Authors:** Boxin Sun, Ming Gu, Amanda Cook Maher, Kelly N. Dubois, Nicholas M Kanaan, Jordan R Bross, Robert Koeppe, Voyko Kavcic, Jian Ren, Tongtong Li, Bruno Giordani

**Affiliations:** ^1^ Michigan State University, East Lansing, MI, USA; ^2^ Michigan Alzheimer's Disease Research Center, Ann Arbor, MI, USA; ^3^ University of Michigan, Ann Arbor, MI, USA; ^4^ Michigan State University, Grand Rapids, MI, USA; ^5^ Wayne State University, Detroit, MI, USA; ^6^ International Institute of Applied Gerontology, Ljubljana, Slovenia

## Abstract

**Background:**

Positron emission tomography (PET) measurement of amyloid in the brain is a widely accepted biomarker for early risk detection of Alzheimer's disease (AD). Blood‐based pTau‐217 is gaining acceptance as a biomarker reflecting both amyloid and tau pathology. In addition, EEG‐based biomarkers are promising in predicting the early stages of neurodegeneration. In this study, we explore the relationship between EEG‐based brain effective connectivity and pTau‐217, and compare their performance in discriminating between Amyloid‐positive and negative participants.

**Method:**

Our research utilizes a dataset with a sample size of n = 22 (7 Amyloid‐positive and 15 Amyloid‐negative, as established by Aβ‐PET Centiloid units, ages 65‐88). All participants were volunteers through the Michigan Alzheimer's Disease Research Center and consensus diagnosed as normal cognition (NC). Each participant was recorded for 3 minutes of eyes‐closed resting‐state EEG (30‐channel), and blood was collected at the same visit to measure levels of plasma pTau‐217 using the SIMOA AlzPath v2 assay.

We first explore the differences in brain effective connectivity between the Amyloid‐positive and Amyloid‐negative groups by applying causalized convergent cross mapping (cCCM) to all the EEG region pairs. Then, relying on machine learning algorithms, we develop a model to predict the Amyloid status using EEG‐based features exploited from brain effective connectivity.

**Result:**

Our study shows that: (i) the Amyloid‐positive group exhibits lower effective connectivity than Amyloid‐negative across certain brain‐region pairs, but higher effective connectivity in others, which may reflect compensatory brain mechanisms; (ii) the cCCM‐based effective connectivity of some EEG region pairs has a strong correlation (>0.6) with pTau‐217; and (iii) for the prediction of Amyloid status, the EEG‐based model is 86.36% accurate, pTau‐217 is 90.91% accurate (with the misclassified participants corresponding to the EEG‐ and pTau‐217‐models being non‐overlapping), and the joint EEG and pTau‐217 model is 95.45% accurate.

**Conclusion:**

Our results indicate that EEG‐based and pTau‐217 biomarkers are highly related and achieve close accuracy in predicting Amyloid status. They also show potential diversity and complementarity in Amyloid status prediction, and both biomarkers could be used jointly to improve the early detection of people at risk of AD.

**Funding**: NSF‐2032709/Li; NIH‐P30AG072931/Paulson; NIH‐P30AG024824/Yung; Maibach‐Smiley‐Alzheimer's‐Research‐Endowment/Kanaan; NIH‐R01AG068338/Giordani, Persad, Murphey.

